# Convention Items

**Published:** 1886-05

**Authors:** 


CONVENTION ITEMS.

PROFESSOR EDWARD SOUCHON, of the Medical Department, Tulane University, (University Louisiana) was a visitor to the

Texas State Medical Association, at Dallas. He expressed himself much interested, as well as surprised at the advanced status of the rank and file of the profession of Texas, with regard to medical lore. The professor favored the association with an able and interesting paper, which we hope to incorporate in the transactions.

ONE OF the most learned and entertaining papers read at the convention was by the distinguished oculist, Dr. B. A. Pope, on "Syphilitic manifestations of the teeth." It will appear in the transactions.

THE COMMITTEE ON PRIZE ESSAYS to award $100 in gold for the best original essay, report nine contestants. The papers are all long, and the committee ask until June 1st, to make the award.

A MOST ENJOYABLE FEATURE of the Dallas meeting was the "receptions," at the houses of resident physicians, held from 3 to 6 o'clock Friday afternoon. There was a grand ball Thursday night; and Wednesday night a superb banquet was spread. We did not attend ; we did not have our speaking outfit with us, and we stayed a little closer off; but we learn there was any amount of toasting and responding.

THE ESSAYIST FOR THE OCCASION, Dr. A. G. Clopton, who was announced to deliver an address for Wednesday evening, disappointed the association. He said he had nevor been notified that he was expected to speak, and the first intimation he had of it, was in this JOURNAL.

NO WINE.—We do not know how such a report ever started, for it has no foundation. We learn that when Austin and Galveston were nominated for the next place of meeting of the Association, and the nominating committee were about to vote on it, it was noised that if they selected Austin, there would be "no banquet and no wine;" and as, strange as it may seem, Austin was elected on that platform. We are not ashamed that we live in a city where abstinence from wine is considered a virtue ; still less ashamed that we belong to a profession to which the absence of wine is an inducement.

A CHAIR OF BIOLOGY IN THE UNIVERSITY OF TEXAS.—Dr. B. E. Hadra, of Austin, introduced the followihg resolution, which was adopted :

*Resolved*, That the Regents of the University of Texas be, and are hereby requested by the State Medical Association, to establish, as soon as practicable, a Chair of Biology in said University, and provide for it all laboratory facilities.

*Resolved*, That Hygiene, which is a branch of Biology, is essential to a finished education, and should be embraced in the curriculum of the University.

*Resolved*. That the facilities to study Bacteriology would not only be of the greatest importance to the physician, but also of the most intrinsic value to the State at large, in discovering the unknown causes of epidemics and the many diseases that at times decimate our herds, and annually embarrass our commercial relations with other States..

EXHIBITS.—As an evidence of the importance of the medical profession of Texas, in the estimation of Manufacturing Chemists and Surgical Instrument Makers, we will state, that the great house of Sharpe & Dohme, Baltimore had an exhibit of their famous chemicals and pharmaceuticals on hand at Dallas, and their polite agent distributed samples with a lavish prodigality. There were also three other chemists represented by displays, and two surgical instrument houses, but as we do not know them "officially," it is not worth while to try to remember their names. "A hint to the wise." The displays were simply elegant, and greater than we ever saw at a State association meeting.

TWO HUNDRED AND FIFTY copies of DANIEL'S MEDICAL JOURNAL, containing the splendid moss-engraving of Dr. Ashbel Smith, were given away at the meeting. They were much sought, and highly prized. We secured a large number of new subscribers, as well as some valuable advertisements.

DR. MORITZ SALM, an oculist, formerly of Austin, now of Atlanta, Ga., was expelled from the State Association for grossly immoral conduct.

DR. H. C. BARNETT, of New Salem, Texas, exhibited at the convention a double-headed monstrosity, delivered by him of a Rusk county multipara.

